# Data in support of comparative physiology and proteomic analysis of two wheat genotypes contrasting in drought tolerance

**DOI:** 10.1016/j.dib.2014.11.001

**Published:** 2014-11-11

**Authors:** Elham Faghani, Javad Gharechahi, Setsuko Komatsu, Mehdi Mirzaei, Ramzan Ali Khavarinejad, Farzaneh Najafi, Laleh Karimi Farsad, Ghasem Hosseini Salekdeh

**Affiliations:** a Department of Systems Biology, Agricultural Biotechnology Research Institute of Iran (ABRII), Karaj, Iran; b National Institute of Crop Science, Kannondai 2-1-18, Tsukuba 305-8518, Japan; c Department of Chemistry and Biomolecular Sciences, Macquarie University, Sydney, NSW, Australia; d Faculty of Science, Kharazmi University of Tehran, Iran; e Department of Molecular Systems Biology at Cell Science Research Center, Royan Institute for Stem cell Biology and Technology, ACECR, Tehran, Iran

## Abstract

Here, we present the data from a comparative physiology and proteomics approach used to analyze the response of two wheat genotypes (SERI M 82 (SE) and SW89.5193/kAu2 (SW)) with contrasting responses to drought stress. Proteomic analysis resulted in identification of 49 unique proteins with significant change in abundance (2-fold) under water shortage in roots and leaves. Gene ontology analysis of drought-responsive proteins (DRPs) suggested an induction of proteins related to cell wall biogenesis, ATP synthesis, photosynthesis, and carbohydrate/energy metabolism in leaves under stress condition. A large fraction of root proteins were identified to be involved in defense and oxidative stress response. In addition, a significant change was detected in proteins related to protein synthesis, ATP synthesis, and germin-like proteins in response to drought stress. A detailed analysis of this data may be obtained from Ref. [1].

## Specifications table

**Table:** 

Subject area	Biology

More specific subject area	Proteomic change associated with drought stress in wheat
Type of data	Protein abundances
How data was acquired	Two-dimensional gel electrophoresis and mass spectrometry
Data format	Normalized data
Experimental factors	Different genotypes (tolerant and sensitive), drought stress, tissue (root or leaf)
Experimental features	Wheat seedlings were subjected to water stress and leaf and root proteome were separated using 2-DE. Differentially abundant proteins in stressed and control plants were identified using nano-LC–MS/MS analysis.
Data source location	Karaj, Iran
Data accessibility	Data is provided in the paper

## Value of the data

• Data provides a combined physiology and proteomic analysis of two wheat genotypes with contrasting responses to drought stress.
• Proteins related to several biological processes including cell wall, oxidative stresses responses, ATP synthesis, photosynthesis and carbohydrate metabolisms were identified to be changed differentially in the tolerant and sensitive genotypes.
• The integrated physiology and proteomic analysis provided a better insight into the molecular responses of wheat plants to drought stress.

## 1 Data, experimental design, materials and methods

Two wheat genotypes (SERI M 82 (SE) and SW89.5193/kAu2 (SW)) were evaluated for drought stress responses at physiology and proteome level. Plant seedlings were grown in PVC pipes and drought stress was imposed by water withholding. Root and leaf samples were collected from stressed and control plants and subjected to 2-DE analysis. In addition, several physiological traits related to water stress including relative water content (RWC), root and shoot dry weight, leaf area, and leaf ABA content were also measured in control and water stressed plants.

## 2 2-Dimensional gel electrophoresis (2-DE)

2-DE was performed as described previously [2]. Gel images were analyzed using the Melanie software (GeneBio, Geneva, Switzerland) as described previously [3]. Spot intensities were subjected to statistical analysis to identify differentially abundant proteins. Only those spots that showed statistical significant differences in roots or leaves of the two tested genotypes and exhibited more than 2-fold change in abundance were accepted as candidate drought-responsive proteins (DRPs). The details of number of reproducibly detected spots and the number of spots showed significant change in roots and leaves of the tolerant (SE) or sensitive (SW) genotypes upon drought stress are shown in Supplementary Table 2.

## 3 Identification of candidate DRPs using mass spectrometry

The candidate DRPs were excised from preparative CBB-stained gels and subjected to the nano-LC–MS/MS analysis. Out of 125 leaf and 112 root protein spots analyzed, 73 and 40 protein species were identified, respectively, representing 49 unique proteins (Supplementary Tables 3 and 4).

## 4 Real time-PCR analysis of the mRNA transcripts of some of the candidate DRPs

To validate the gene expression of some of the candidate DRPs, we further applied quantitative real time-PCR analysis (Supplementary Table 1). Overall, our result showed that there is no clear correlation between the mRNA expression and the protein abundance estimated from spot densities.
