# Supplementary material for: Data in support of comparative physiology and proteomic analysis of two wheat genotypes contrasting in drought tolerance
Source: Data Brief. 2014 Nov 11;2:26–8. doi: 10.1016/j.dib.2014.11.001 (PMC4459561; doi:10.1016/j.dib.2014.11.001)
Supplement: Supplementary file 1 — Supplementary data [file mmc1.doc]

**Supplementary table 1**: The sequence of the primers used for real time-PCR analysis of some of the candidate DRPs.

| Tissue | Gene name | Primer sequences | Product length |
| --- | --- | --- | --- |
| Root | 16.9 kDa class I heat shock protein 1 (16.9 kDa HSP) | Fw: AGAAACCACACACACACGAGTC  Re: AGAAGCCCGAGGTGAAGAG | 123 |
| Root | Glutathione-S-transferase (GST) | Fw: GACATCCTCAAGACCCTCGAC  Re: CCTCTCGTAGCTGTGGAACC | 120 |
| Leaf | Glyceraldehyde-3-phosphate dehydrogenase (GA3PD) | Fw: ATTGAGGGTTTGATGACCACTG  Re: GCTGCTGGGAATGATGTTAAAG | 120 |
| Leaf  Root | Cu/Zn superoxide dismutase (SOD) | Fw: GGGTGCATATCAACAGGTCC  Re: CGCCACACCTTCAGCATTG | 120 |
| Leaf | Ribulose-1,5-bisphosphate carboxylase/oxygenase LS (RUBISCO) | Fw: TGCAGCAGCTAATCGAGTGG  Re:GCTAGTTCAGGACTCCATTTGC | 120 |
| Root | Ascorbate peroxidase (APX) | Fw: TTTGACGGTGCATGGACTCG  Re: GCGTCGAAATTCAGGATCATCC | 135 |
| Leaf | Protein disulfide isomerase (PDI) | Fw: GCCACTTACCTTGAAGACGG  Re: GCCAAAGTCATAATCAGACCG | 120 |
| Root | ATP synthase beta subunit (ATPB) | Fw: TCTGAGGTGTCTGCTCTCCTTG  Re: CCCTTCTTTGTCGTCGTAATCC | 110 |
| Leaf | ATP synthase alpha subunit (ATPA) | Fw: TCGCAGCATCAAGGTCTGAC  Re: GCCGCTCAGTTGAAAGCTATG | 112 |
| Leaf | Phosphoglycerate kinase (PGK) | Fw: TTGAAGCCTTCTGTTGCTGG  Re: CTTGGATGACACCTTTGAGCC | 120 |
| Housekeeping  gene | 18S ribosomal RNA gene (18S rRNA) | Fw: TTAACGAACGAGACCTCAGCC  Re: GGCATGACAGACCTGTTATTGC | 124 |

**Supplementary table 2**: number of reproducibly detected spots in 2-DE gels of roots and leaves of the two tested genotypes along with the number of spots which showed significant change to drought stress and number of proteins that could be successfully identified using nano-LC-MS/MS analysis. In most of the cases differentially abundant proteins showed significant change in both genotypes and tissues.

| Number of spots | Genotype | | | |
| --- | --- | --- | --- | --- |
|  | Tolerant (SE) | | Sensitive (SW) | |
|  | Tissue | | Tissue | |
|  | Root | leaf | root | leaf |
| Total number of reproducibly detected spots | 730 | 885 | 730 | 885 |
| Number of spot with significant change | 118 | 101 | 127 | 121 |
| Number of spot subjected to MS analysis | 95 | 83 | 69 | 89 |
| Number of identifies proteins | 29 | 48 | 31 | 51 |

**Supplementary Table 3:** Differentially accumulated proteins in leaves of the two wheat genotypes (Tolerant (SE) and Sensitive (SW)) identified using nano LC-ESI-MS/MS during drought stress. Asterisk indicates that the identified protein is the top hit (higher score) entry of the multiply identified proteins detected in the corresponding spot (Table 2).

| Spot ID^a^ | MW/pI^b^ | | Score/%Cov^c^ | Protein identity/Accession number | NMP^d^ | Fold change in abundance | |
| --- | --- | --- | --- | --- | --- | --- | --- |
|  | Exp | The |  |  |  | Sensitive | Tolerant |
| **Cell wall biogenesis and degradation** | | | | | | | |
| 325L | 31/5.6 | 34.9/5.6 | 271/23.4 | Beta-1,3-glucanase/Q9XEN5 | 9 | 3.57 | 2.4 |
| 400L | 27/4.97 | 34.9/5.6 | 87/10.2 | Beta-1,3-glucanase/Q9XEN5 | 3 | 3.68 | 1.4 |
| 476L | 33/4.83 | 34.9/5.6 | 47/6.6 | Beta-1,3-glucanase/Q9XEN5 | 2 | -1.29 | 2 |
| 562L | 33/5.6 | 34.9/5.6 | 100/16.2 | Beta-1,3-glucanase/Q9XEN5 | 4 | 2.74 | 1.61 |
| 440L | 32/5.3 | 34.9/5.6 | 117/11.1 | Beta-1,3-glucanase/Q9XEN5 | 4 | -2 | -2.5 |
| 613L٭ | 42/4.56 | 36.2/4.5 | 165/29.8 | Endo-beta-1,3-glucanase/Q1ERF7 | 7 | -1.44 | -2 |
| 612L | 43/4.5 | 36.2/4.5 | 71/8.2 | Endo-beta-1,3-glucanase/Q1ERF7 | 2 | 2.68 | 1.1 |
| 615L | 41/4.6 | 35.9/4.7 | 100/14.1 | Endo-beta-1,3-glucanase/Q1ERG1 | 6 | -9.09 | -2 |
| 614L | 42/4.6 | 35.9/4.7 | 140/19.1 | Endo-beta-1,3-glucanase/Q1ERG1 | 3 | -4.54 | -5.2 |
| **Defense and oxidative stress** | | | | | | | |
| 54L | 18/5.32 | 20.3/3.3 | 378/52.2 | Superoxide dismutase [Cu-Zn]/O24400 | 14 | -2 | -1.16 |
| 110L | 33/4.34 | 20.4/5.3 | 83/32.8 | Superoxide dismutase [Cu-Zn]/Q96123 | 3 | -4 | -2.17 |
| 297L | 28/5.8 | 20.3/5.3 | 52/31.8 | Superoxide dismutase [Cu-Zn]/O24400 | 3 | 2.16 | 1.2 |
| 446L٭ | 29/5.1 | 26.7/5.5 | 156/27.2 | Ascorbate peroxidase/ C3VQ52 | 7 | 1.75 | 2.04 |
| 159L | 26/6.5 | 24.9/5.9 | 88/11.5 | Putative glutathione S-transferase/Q9FUD8 | 2 | -2.70 | 1.94 |
| 356L٭ | 26/5.34 | 28.1/6.3 | 246/37.6 | Thioredoxin peroxidase /O81480 | 8 | -2.7 | -1.1 |
| 434L | 26/5.06 | 28.1/6.3 | 206/37.6 | Thioredoxin peroxidase /O81480 | 5 | -1.11 | -2 |
| 473L | 32/4.9 | 29.0/7.7 | 76/11.3 | Harpin binding protein 1/Q5QJA5 | 3 | 4.7 | 1.8 |
| **Protein synthesis and processing** | | | | | | | |
| 678L | 73/4.97 | 56.9/4.9 | 64/7.4 | Protein disulfide isomerase/B9A8E2 | 3 | 2 | 1.07 |
| 472L | 29/5.02 | 16.1/10.3 | 143/38 | 60S ribosomal protein L13/Q8S3J1 | 7 | -3.84 | 1.73 |
| 406L | 28/4.6 | 26.0/4.6 | 425/35.9 | Proteasome subunit alpha type/I1IKE1 | 8 | 2.2 | -1.63 |
| 33L | 14/5.5 | 12.2/6.5 | 170/30.4 | Peptidyl-prolyl isomerase /M7Z599 | 4 | -2.5 | -2 |
| 346L | 29/5.5.8 | 15.1/10.4 | 89/12.9 | Ribosomal protein L17/Q5I7K4 | 3 | -4.16 | 1.08 |
| 326L | 32/5.6 | 15.1/10.4 | 186/12.9 | Ribosomal protein L17/Q5I7K4 | 4 | -2.38 | 1.04 |
| 569L | 36/5.51 | 34.1/5.63 | 134/17.5 | Cysteine synthase/M8CF13 | 4 | -1.12 | -2 |
| 124L | 27/4.18 | 43.6/5.5 | 71/5.1 | S-adenosylmethionine synthase/B0LXM0 | 2 | -3.44 | 1.32 |
| 795L | 58/5.92 | 43.6/5.5 | 196/19.9 | S-adenosylmethionine synthase/B0LXM0 | 6 | -2 | 1.38 |
| 704L | 40/5.06 | 43.6/5.5 | 70/5.3 | S-adenosylmethionine synthase/B0LXM0 | 2 | -1.13 | 2.11 |
| **Carbohydrate and energy metabolism** | | | | | | | |
| 332L | 28/5.6 | 27.0/5.3 | 433/47.4 | Triosephosphate isomerase /Q9FS79 | 16 | 2.06 | 1.32 |
| 218L | 42/6.24 | 36.6/6.6 | 150/11 | Glyceraldehyde-3-phosphate dehydrogenase/C7C4X1 | 3 | 3.04 | 1.5 |
| 304L | 32/5.9 | 26.2/7.6 | 119/19.5 | Adenylate kinase A/M8CA41 | 5 | -2.56 | 1.8 |
| 583L | 39/5.1 | 42.2/5.9 | 65/12.6 | Fructose-bisphosphate aldolase/C0KTA6 | 3 | -2.43 | 1.58 |
| 591L | 41/5.1 | 42.2/5.9 | 73/8.2 | Fructose-bisphosphate aldolase/C0KTA6 | 2 | -2.27 | 2.02 |
| 539L٭ | 40/5.6 | 42.2/5.9 | 803/39.4 | Fructose-bisphosphate aldolase/C0KTA6 | 23 | 4.92 | 1.48 |
| 708L | 40/5.26 | 42.2/5.9 | 243/31.4 | Fructose-bisphosphate aldolase/C0KTA6 | 12 | 2.09 | 5.8 |
| 545L | 37/5.6 | 42.2/5.9 | 85/12.1 | Fructose-bisphosphate aldolase/C0KTA6 | 3 | -3.12 | -2 |
| 513L٭ | 42/6.04 | 35.8/5.7 | 418/40.5 | Malate dehydrogenase/ | 12 | -5.55 | 1.9 |
| 805L | 19/5.48 | 19.8/5.0 | 187/9.9 | Adenine phosphoribosyltransferase/Q43199 | 3 | -2.7 | -3.57 |
| 812L | 22/5.7 | 21.9/5.6 | 100/15.6 | Adenosine diphosphate glucose pyrophosphatase/Q8L686 | 2 | -2.5 | -2.32 |
| 271L | 22/6 | 21.9/5.6 | 147/15.6 | Adenosine diphosphate glucose pyrophosphatase/Q8L686 | 5 | -1.49 | -5.88 |
| 429L٭ | 26/5.02 | 19.8/5.0 | 414/49.7 | Adenine phosphoribosyltransferase/Q43199 | 10 | 1.2 | 5.09 |
| 340L | 28/5.5 | 24.4/5.4 | 119/37.4 | inorganic pyrophosphotase/AFK26595 | 6 | 2.24 | 1.3 |
| 747L | 57/5.1 | 43.0/5.3 | 107/5.1 | Gamma-glutamylcysteine synthetase/Q5G1T9 | 2 | 1.55 | 2.44 |
| 621L٭ | 43/4.5 | 45.5/5.7 | 224/18.6 | Phosphoribulokinase, chloroplastic/P26302 | 6 | 3.56 | 2.9 |
| 735L٭ | 44/5.34 | 45.5/5.7 | 445/34.7 | Phosphoribulokinase, chloroplastic/P26302 | 14 | -1.63 | 2 |
| 848L | 50/4.94 | 49.9/6.5 | 262/31.9 | Phosphoglycerate kinase, chloroplastic/P12782 | 7 | 1.63 | -3.57 |
| **ATP synthesis** | | | | | | | |
| 76L | 15/4.8 | 15.2/5.2 | 107/31.4 | ATP synthase epsilon chain, chloroplastic/P69445 | 2 | 4.44 | -1.69 |
| 734L٭ | 42/5.2 | 40.0/8.1 | 222/14 | ATP synthase gamma chain/D3K4D8 | 7 | -2.43 | 1.55 |
| 329L | 29/5.6 | 27.0/7.7 | 349/37.8 | ATP synthase, mitochondrial/Q6IY71 | 13 | 2 | 1.33 |
| 361L٭ | 30/5.2 | 55.5/5.7 | 314/15.5 | ATP synthase subunit alpha, mitochondrial /Q332R4 | 5 | -2 | 1.06 |
| 824L٭ | 32/5.5 | 55.5/5.7 | 201/8.4 | ATP synthase subunit alpha, mitochondrial/P12862 | 2 | 2 | -1.44 |
| 815L٭ | 25/5.8 | 55.5/5.7 | 190/11.2 | ATP synthase subunit alpha, mitochondrial/P12862 | 3 | -2.27 | -1.01 |
| 810L٭ | 21/5.6 | 55.5/5.7 | 217/11.2 | ATP synthase subunit alpha, mitochondrial/P12862 | 3 | 3.58 | -1.26 |
| 24L | 18/5.7 | 24110/8.4 | 138/14.9 | Cytochrome b6-f complex iron-sulfur subunit, chloroplastic/Q7X9A6 | 4 | 5.98 | -1.61 |
| 588L | 38/5.02 | 40.4/6.9 | 157/16.5 | Ferredoxin-NADP(H) oxidoreductase/Q8RVZ8 | 4 | -1.06 | 2.1 |
| **Photosynthesis** | | | | | | | |
| 294L٭ | 26/5.9 | 27.4/8.8 | 869/52.7 | Oxygen-evolving enhancer protein 2, chloroplastic/Q00434 | 24 | 2 | 1.07 |
| 230L | 38/6.2 | 27.4/8.8 | 252/23.6 | Oxygen-evolving enhancer protein 2, chloroplastic/Q00434 | 6 | -2.12 | -1.11 |
| 162L | 27/6.43 | 27.4/8.8 | 129/8.1 | Oxygen-evolving enhancer protein 2, chloroplastic/Q00434 | 3 | -1.17 | -2.22 |
| 265L٭ | 26/5.9 | 27.4/8.8 | 158/15.9 | Oxygen-evolving enhancer protein 2, chloroplastic/Q00434 | 4 | -7.69 | 1.7 |
| 269L | 25/6.05 | 27.4/8.8 | 397/5 | Oxygen-evolving enhancer protein 2, chloroplastic/Q00434 | 9 | -1.07 | -2.08 |
| 221L٭ | 40/6.3 | 22.4/4.9 | 274/38.8 | Ribulose-1,5-bisphosphate carboxylase activase/Q6XW16 | 9 | -1.85 | 2.46 |
| 319L | 30/5.7 | 22.4/4.9 | 283/38.8 | Ribulose-1,5-bisphosphate carboxylase activase/Q6XW16 | 10 | 2.23 | 1.36 |
| 843L | 61/6.3 | 53.6/6.2 | 2749/62.2 | Ribulose bisphosphate carboxylase large chain/P05698 | 110 | 5.38 | 1.19 |
| 846L | 56/5.03 | 53.6/6.2 | 431/28.2 | Ribulose bisphosphate carboxylase large chain/P05698 | 23 | -3.44 | 1.01 |
| 582L | 37/5.2 | 53.6/6.2 | 288/35.3 | Ribulose bisphosphate carboxylase large chain/ P05698 | 18 | -4.54 | 3.13 |
| 727L٭ | 66/5.4 | 53.6/6.2 | 174/14.6 | Ribulose bisphosphate carboxylase large chain/P05698 | 5 | 1.55 | -2.17 |
| 19L٭ | 17/5.9 | 19.7/8.8 | 715/62.3 | Ribulose bisphosphate carboxylase small chain/Q9FRZ4 | 31 | 2 | 1 |
| 266L | 24/6.05 | 19.7/8.8 | 348/64 | Ribulose bisphosphate carboxylase small chain/Q9FRZ4 | 21 | 1.55 | 2 |
| 711L | 59/4.9 | 19.7/8.5 | 107/21.7 | Ribulose bisphosphate carboxylase small chain/Q9FRZ4 | 2 | -9.09 | 1.63 |
| 272L٭ | 21/6 | 19.7/8.5 | 270/34.3 | Ribulose bisphosphate carboxylase small chain/Q9FRZ4 | 6 | -2 | -3.12 |
| **Unclassified proteins** | | | | | | | |
| 816L | 25/5.64 | 19.3/5.6 | 67/26 | Basic transcription factor 3/K4MRD6 | 3 | -3.7 | -3.70 |
| 420L | 29/4.9 | 18.9/4.7 | 263/47.1 | Cp31BHv/C3V134 | 7 | 2.76 | 1.71 |
| 411L | 30/4.5 | 18.9/4.8 | 216/37.6 | Cp31BHv/C3V134 | 5 | -2.12 | 1.81 |
| 102L٭ | 22/4.1 | 13.4/4.4 | 236/56.3 | unnamed protein product/CAX04390 | 7 | -2.70 | 1.94 |

1. The numbering corresponds to match ID in 2-DE gels
2. The theoretical (The) and experimental (Exp) molecular weight (MW, kDa) and isoelectric point (pI)
3. Nano LC-MS score (Score) and percent of sequence coverage (%Cov)
4. Number of matched peptide (NMP)

**Supplementary Table 4**: Differentially accumulated proteins in roots of the two wheat genotypes (Tolerant (SE) and Semsitive (SW)) identified using nano LC-ESI-MS/MS during drought stress. Asterisk shows the top hit entry (higher score) from the list of the identified proteins in the corresponding spot (Table 2).

| Spot ID^a^ | MW/pI^b^ | | Score/%Cov^c^ | Protein identity/Accession number | NMP^d^ | Fold change in abundance | |
| --- | --- | --- | --- | --- | --- | --- | --- |
|  | Exp | The |  |  |  | Sensitive | Tolerant |
| **Carbohydrate metabolism** | | | | | | | |
| 423R٭ | 32/6.2 | 29.4/8.7 | 179/13.9 | Fructose-bisphosphatealdolase/Q8VWM9 | 5 | -1.16 | -2.43 |
| 400R | 34/6.2 | 29.4/8.7 | 121/13.5 | Fructose-bisphosphatealdolase/Q8VWM9 | 4 | -2.5 | 1.32 |
| 310R٭ | 40/5.7 | 39.1/6.8 | 150/28.2 | Malate dehydrogenase/A3KLL4 | 6 | -1.42 | 2.13 |
| 182R | 27/4.9 | 19.8/5.0 | 202/26 | Adenine phosphoribosyltransferase/Q43199 | 7 | -1.31 | -2.04 |
| 410R٭ | 34/6.1 | 35.1/5.96 | 140/6.6 | Glucan endo-1,3-beta-glucosidase/D8L9Q2 | 2 | -2.08 | 1.51 |
| 691R | 42/6.1 | 64.1/5.6 | 101/7.3 | Beta-glucosidase/D5MTF8 | 2 | 1.77 | 2.06 |
| **Defense and oxidative stress** | | | | | | | |
| 90R | 30/6.3 | 24.8/5.9 | 164/24.9 | Germin-like protein/Q9SM34 | 6 | -1.11 | 2.29 |
| 106R | 30/6.01 | 24.8/5.9 | 91/8.3 | Germin-like protein/Q9SM34 | 2 | 2.01 | 1.43 |
| 337R | 26/5.5 | 23.8/6.4 | 52/11.2 | Germin-like protein/C3UZE6 | 2 | 2.02 | 1.72 |
| 83R | 30/6.4 | 24.8/5.9 | 103/17.9 | Germin-like protein/ Q9SM34 | 3 | -1.05 | 2.09 |
| 92R٭ | 30/6.2 | 24.8/5.9 | 345/36.2 | Germin-like protein/Q9SM34 | 12 | -1.41 | 2 |
| 129R | 21/5.8 | 19.5/5.6 | 242/21 | Cold shock domain protein/Q75QN9 | 5 | 2.02 | 1.01 |
| 10R | 18/5.08 | 17.1/5.2 | 108/60.6 | Pathogenesis-related protein/H2KXF7 | 7 | 3.07 | 1 |
| 4R | 20/5.9 | 17.1/5.2 | 83/14.4 | Pathogenesis related protein 10/B5B3P8 | 6 | -2.12 | -2.17 |
| 269R | 19/5.12 | 20.4/5.3 | 240/52.2 | Superoxide dismutase [Cu-Zn]/Q96123 | 7 | 3.41 | 2.31 |
| 109R٭ | 30/6.06 | 26.7/5.5 | 204/38.7 | Ascorbate peroxidase/C3VQ52 | 13 | -1.42 | 2.24 |
| 392R | 38/6.3 | 36.9/5.5 | 125/20.2 | Peroxidase/Q5GMP4 | 6 | 1.68 | 4.9 |
| 220R | 37/6.5 | 36.9/5.5 | 465/51.9 | Peroxidase/Q5GMP4 | 17 | -2.08 | -1.33 |
| 111R | 31/6.01 | 23.7/5.9 | 106/9.4 | Glutathione transferase/Q8GTB7 | 2 | -2.04 | 1.49 |
| 86R٭ | 28/6.3 | 25.1/6.3 | 427/37.4 | Glutathione S-transferase/Q8RW02 | 16 | -1.63 | 5.58 |
| 25R | 21/5.1 | 25.1/6.3 | 74/17.6 | Glutathione S-transferase/Q8RW02 | 2 | -1.13 | 4.44 |
| 304R | 28/6.08 | 25.1/6.3 | 143/17.6 | Glutathione S-transferase/Q8RW02 | 3 | 2.2 | 6.19 |
| **ATP synthesis** | | | | | | | |
| 656R | 61/5.2 | 59.3/5.5 | 568/35.4 | ATP synthase subunit beta/Q41534 | 14 | -2.32 | 1.18 |
| 651R | 59/5.1 | 59.3/5.5 | 39/10.3 | ATP synthase subunit beta/Q41534 | 3 | -1.88 | -3.44 |
| 650R٭ | 59/4.9 | 59.3/5.5 | 268/18.6 | ATP synthase subunit beta/Q41534 | 7 | -3.12 | -2 |
| **Protein synthesis and processing** | | | | | | | |
| 147R | 27/5.3 | 49.4/9.2 | 106/7.6 | Elongation factor 1-alpha/Q03033 | 3 | -2.5 | -2.56 |
| 139R | 27/5.31 | 49.4/9.2 | 144/16.8 | Elongation factor 1-alpha/Q03033 | 5 | 2.33 | 1.14 |
| 146R | 26/5.06 | 49.4/9.2 | 159/14.1 | Elongation factor 1-alpha/Q03033 | 5 | -1.13 | -2.04 |
| 679R | 70/5.04 | 56.6/4.9 | 267/30.1 | Protein disulfide isomerase/F8THZ7 | 10 | -2.32 | -2.12 |
| 677R | 72/4.9 | 56.6/5.0 | 48/10.5 | Protein disulfide isomerase/B9A8E3 | 4 | -4.34 | -2.22 |
| 676R | 72/4.9 | 56.9/4.9 | 205/17.3 | Protein disulfide isomerase/B9A8E2 | 7 | -2.32 | -3.03 |
| 680R | 73/4.8 | 56.9/4.9 | 95/12 | Protein disulfide isomerase/B9A8E2 | 4 | -3.57 | -5.88 |
| 681R | 74/4.8 | 56.9/4.9 | 81/7.8 | Protein disulfide isomerase/B9A8E2 | 3 | 1.2 | -5.55 |
| 275R | 18/5.5 | 68.65.8 | 157/22.5 | 16.9 kDa class I heat shock protein/P12810 | 3 | 3.32 | 2.69 |
| 287R | 21/5.6 | 16.8/5.8 | 144/22.5 | 16.9 kDa class I heat shock protein/P12810 | 3 | 2.14 | 1.65 |
| 48R | 21/6.2 | 16.8/5.8 | 84/13.2 | 16.9 kDa class I heat shock protein/P12810 | 2 | 2.1 | 4.83 |
| 141R | 21/5.01 | 16.8/5.8 | 82/22.5 | 16.9 kDa class I heat shock protein/P12810 | 2 | 1.02 | 2 |
| 327R٭ | 18/5.5 | 17.6/5.9 | 186/44.4 | 17.6kDa heat-shock protein/A5A8T7 | 8 | 6.56 | 3.03 |
| **Signal transduction** | | | | | | | |
| 567R | 36/4.3 | 29.3/4.8 | 116/12.3 | 14-3-3 protein/Q08G36 | 4 | -10 | 1.75 |
| 570R | 44/4.4 | 29.3/4.8 | 126/42.1 | 14-3-3 protein/Q08G36 | 7 | -2.22 | -1.53 |

1. The numbering corresponds to match ID in 2-DE gels
2. The theoretical (The) and experimental (Exp) molecular weight (MW, kDa) and isoelectric point (pI)
3. Nano LC-MS score (Score) and percent of sequence coverage (%Cov)
4. Number of matched peptide (NMP)
